# Transition to practice: creation of a transitional rotation for radiation oncology

**Published:** 2018-07-27

**Authors:** Hannah Dahn, Karen Watts, Lara Best, David Bowes

**Affiliations:** 1 Department of Radiology Oncology, Dalhousie University, Nova Scotia, Canada

## Abstract

**Background:**

Implementation of Competence by Design (CBD) will require residency training programs to develop formalized “Transition to Practice” (TTP) experiences. A multidisciplinary group of Radiation Oncology stakeholders from tertiary care centres in Atlantic Canada were surveyed regarding a proposed TTP rotation.

**Methods:**

The survey asked participants to quantitatively rank various learning objectives based on defined CanMEDS skills that are expected to be mastered by a graduating resident. Mean perceived importance scores were calculated for each objective as well as for their CanMEDS category. Specific written qualitative feedback was also collected.

**Results:**

The survey was circulated to 59 participants with a response rate of 73%. The three objectives with the highest mean importance score were “Independently assessing and managing patients seen in consultation,” “Developing and demonstrating communication skills with patients at an advanced level,” and “Independently assessing and managing follow up patients,” respectively from highest to lowest. The CanMEDS roles with the highest importance score was “Communicator.”

**Conclusion:**

Quantitative and qualitative data from a multidisciplinary survey based on CanMEDS roles guided the implementation of a TTP rotation for PGY-5 residents at a tertiary care centre in Atlantic Canada. These results may be relevant to other training programs developing TTP experiences.

## Introduction

The transition from residency to working as an attending staff is a significant stage in any physician’s career. New attending physicians are faced with potentially unfamiliar tasks, including having the final responsibility for patient care, taking on supervisory roles and performing managerial or administrative duties.^1^ Many new attending physicians feel more confident with their clinical knowledge than with their non-clinical skills.^2,3^ Due to this, they experience more stress when dealing with non-clinical issues that fall outside of the Medical Expert role, such as managing finances, meeting performance targets, resolving conflict with colleagues and maintaining a healthy work-life balance.^4^

In Radiation Oncology, an attending physician must perform many tasks to which residents may not have had exposure during training, such as having final responsibility for independently assessing and treating patients, hiring support staff, developing treatment protocols and chairing departmental or multi-disciplinary meetings. A survey of Canadian Radiation Oncology residents in 2009 showed that 35.8% of respondents planned on doing a fellowship with the goal of obtaining more training, in both clinical and non-clinical skills, in order to ease the transition to practice.^5^

Residency programs across Canada are implementing the Royal College of Physicians and Surgeons of Canada’s (RCPSC) Competence by Design (CBD) framework which focuses on deliberate, competency based teaching and assessment through four stages: Transition to Discipline, Foundations of Discipline, Core of Discipline, and Transition to Practice (TTP).^6^ Residents entering the TTP stage will already have demonstrated competence in the “core” skills of the specialty, and the intention is to provide residents with the remaining skills required to enter autonomous practice.

Example TTP rotations have been described in the literature ranging from a weekend retreat focused on business-of-medicine education for anesthesia residents to year-long operative autonomy and practice management experiences for surgical residents.^7-9^ The Department of Anesthesia at the University of Ottawa launched a Competence by Design (CBD) program in 2015 with a TTP rotation that included task checklists and daily oral examinations.^10^ A collection of Emergency medicine program directors suggest that newly appointed physicians focus on a number of non-clinical skills as they begin practice including personal activities outside of work, finances, compassionate care, preventing burnout, interpersonal interactions, and professional development.^11^ Also, for engaging in complex patient interactions, residents need advanced communication skill training consisting of lectures and deliberate practice with simulated patients.^12,13^

In anticipation of transitioning to CBD, our centre is developing a formalized TTP rotation for residents in their last three months of training. Content development can be informed by consulting healthcare members, educators and students.^14,15^ A survey was sent to the relevant stakeholders across Atlantic Canada, regarding the relative importance of learning objectives, to guide curriculum development for the proposed TTP rotation. The purpose of this study was to guide the creation of a TTP rotation at our centre, which exposed residents to clinical and non-clinical skills in which they may not yet have become competent. This study may also inform the development of TTP experiences in other specialty training programs at other centres.

## Methods

A voluntary, anonymous online survey was created using Google Forms. Nova Scotia Health Authority Research Ethics Board waived the need for ethics approval. The survey was circulated via email to all current departmental faculty (including Radiation Oncologists, Medical Physicists and Radiation Therapists), Radiation Oncology residents who graduated in the previous five years, current and former residency program committee members (including representation from nursing, medical physics and radiation therapy), and other Radiation Oncologists in the Atlantic region who did not meet the above criteria. The survey asked participants to quantitatively score selected learning objectives chosen from our current program objectives for senior residents and reflecting the CanMEDS skills that are expected to be mastered by a graduating resident, using the Royal College Objectives of training as a reference.^16^ Mean scores (from 0: not important to 10: extremely important) and standard deviations were calculated for each objective as well as mean scores for pooled objectives based on their CanMEDS category. The minimum and maximum mean objective scores and pooled CanMEDS category scores were compared using Student’s and Welch’s t-test for unequal sample sizes. Qualitative feedback was collected by asking what specific objectives should be included in the rotation, if there were any specific ideas for potential projects to complete during the rotation, and how the rotation should be evaluated. Respondents were also given the opportunity to provide free-form feedback and comments. Thematic analysis was performed by two separate authors by examining the raw free-form responses and assigning descriptive codes which were then grouped into themes that were agreed upon by all authors. Themes and meaningful unique responses were reported.

## Results

The survey was circulated to 59 participants with a response rate of 73% (n=43). Responses came from Nova Scotia (26), New Brunswick (7), Prince Edward Island (1), Newfoundland (5), and other (4). Respondents included Radiation Oncologists (30), Radiation Oncology Residents (4), Medical Physicists (4), Radiation Therapists (2), Nursing (2), and other (1).

**Figure 1:**
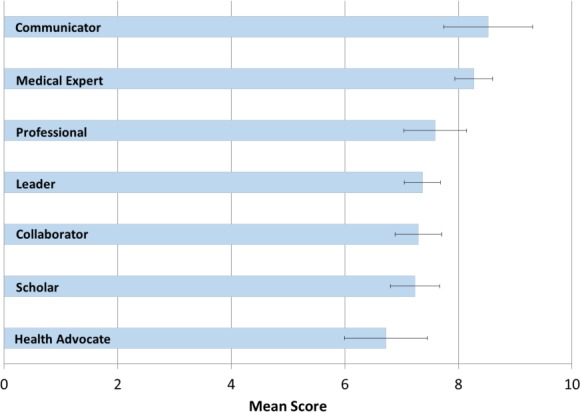
Mean ranks and standard deviations for pooled CanMEDS objective categories from 0 (not important) to 10 (extremely important)

The mean scores and standard deviations for the perceived importance of the learning objectives were calculated (Table 1). The three objectives with the highest mean rank score were “Independently assessing and managing patients seen in consultation,” “Developing and demonstrating communication skills with patients at an advanced level,” and “Independently assessing and managing follow up patients,” with scores of 9.2, 8.5 and 8.4 respectively. The three objectives with the lowest mean scores were “Completing a research, administrative, educational, or clinical project,” “Health advocacy topics,” and “Working with departmental and hospital administration,” with scores of 6.6, 6.7 and 6.7 respectively. There is a significant difference between 9.2 and 6.6 using Student’s t-test (p=0.001). The learning objectives were then grouped into CanMEDS categories, based on the original RCPSC objectives, and their summed mean scores and standard deviations were calculated (Figure 1). Objectives that represented a single CanMEDS role were given a weight of 1 and objectives that represented two CanMEDS roles were given a weight of 0.5 in both summed CanMEDS calculations. The summed CanMEDS roles with the highest and lowest mean scores were “Communicator” with a score of 8.5 and “Health Advocate” with a score of 6.7. There is a significant difference between these means using Welch’s t-test (p = 0.03).

**Table 1:** Mean ranks and standard deviations for candidate learning objectives on a scale from 0 (not important) to 10 (extremely important)

CanMEDS objective	Mean	SD

Independently assessing and managing patients seen in consultation (Medical Expert)	9.2	+/- 0.7
Independently assessing and managing follow up patients (Medical Expert)	8.4	+/- 0.7
Specialized knowledge in specific tumour sites or techniques (Medical Expert)	7.8	+/- 1.0
Triaging and managing outside calls and emergencies arising during daytime hours (Medical Expert/Collaborator)	8.2	+/- 0.6
Managing inpatients with increased responsibility (Medical Expert/Collaborator)	7.4	+/- 0.6

Developing skills in conflict resolution (Collaborator)	7.1	+/- 0.5
Chairing tumour boards, quality assurance rounds, and other clinical meetings (Collaborator)	7.0	+/- 0.7

Developing and demonstrating communication skills with patients at an advanced level (Communicator)	8.5	+/- 0.7

Independently dealing with managerial and administrative aspects of a clinical practice (Leader)	7.9	+/- 0.6
Developing leadership skills (Leader)	7.5	+/- 0.6
Participating in patient safety initiatives (Leader)	7.3	+/- 0.7
Working with departmental and hospital administration (Leader)	6.7	+/- 0.7

Building a relationship with a mentor(s) (Professional)	7.5	+/- 0.7

Developing a long term plan for learning and professional development (Professional/Scholar)	7.8	+/- 0.7

Teaching other trainees and health professionals (Scholar)	7.6	+/- 0.6
Completing a research, administrative, educational, or clinical project (Scholar)	6.6	+/- 0.4

Health advocacy topics (Health Advocate)	6.7	+/- 0.8

**Figure 2:**
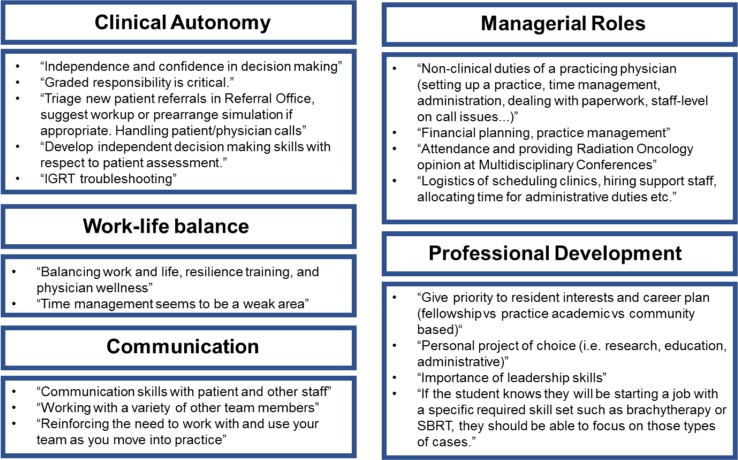
Summarized qualitative data showing common themes for suggested specific TTP rotation objectives. (IGRT = image guided radiotherapy; SBRT = stereotactic body radiotherapy)

**Figure 3:**
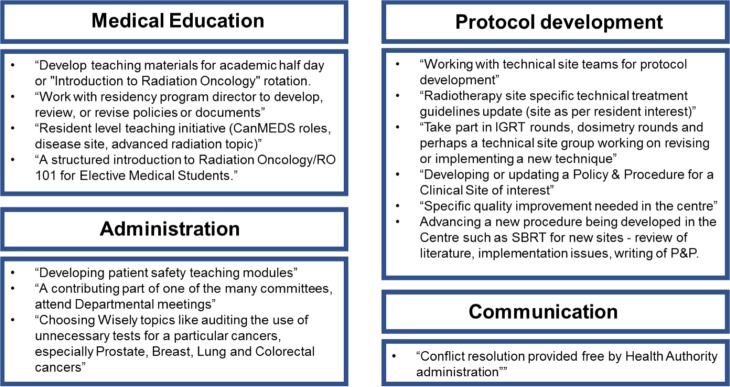
Summarized qualitative data showing common themes for suggested independent TTP rotation projects

**Figure 4:**
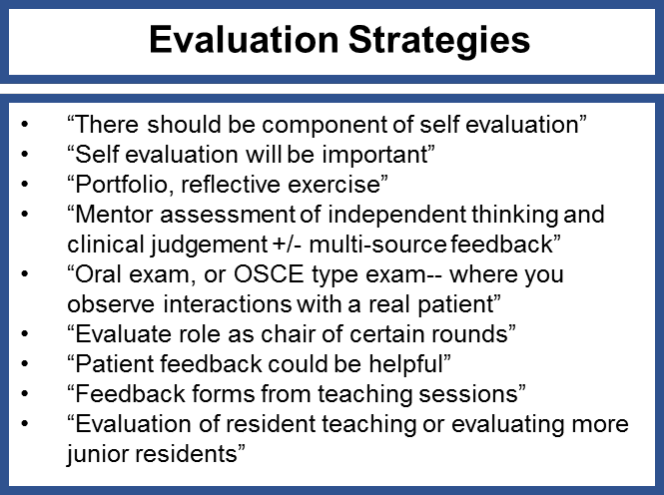
Summarized qualitative data showing suggested TTP rotation evaluation strategies

Qualitative data were collected to gather detailed suggestions for the TTP rotation. Respondents were asked to elaborate on what objectives should be included in the rotation. Common themes emerged including a suggested focus on managerial roles, clinical autonomy, work-life balance, professional development, and communication (Figure 2). Independent project suggestions involved topics in medical education, protocol development, communication and administration (Figure 3). Suggested strategies for evaluation of the TTP rotation included self-evaluation, formal testing using daily feedback, oral exams and OSCE-style exams as well as evaluations by patients, students and committee members (Figure 4).

## Discussion

This survey has provided information about how a TTP rotation could be structured to improve the transition to independent practice in Radiation Oncology. In this survey, the CanMEDS roles which were most highly prioritized by respondents were Communicator and Medical Expert. However, when analyzing the qualitative data from suggested TTP rotation objectives, the common themes varied to include other areas of importance such as clinical autonomy, managerial roles, professional development, and work-life balance. The Communicator CanMEDS role was ranked highly which likely represents the high priority placed on advanced communication skills by the survey respondents. Strong physician communication skills have been shown to be associated with increased patient satisfaction and better health outcomes.^17^ In addition, there is evidence that many residents struggle with complex communication skills and perhaps this role was ranked highly as effective communication is central to being a Radiation Oncologist.^18,19^

No respondents suggested that time be spent furthering one’s medical knowledge; this likely reflects the fact that it would be expected that residents entering such a rotation will already have demonstrated the appropriate medical knowledge required to practice independently. Respondents were informed that this rotation would take place around and after the time during which residents complete their certification examinations, and during this time it is expected that residents would transition out of the phase where there is a strong priority placed on the Medical Expert domain. Literature shows that residents nearing the end of their training feel very confident with their clinical knowledge.^20-22^ A study of 46 newly appointed consultants found that 96% of them felt prepared or very well prepared for the clinical aspects of their new role.^23^ A survey of 1228 recently graduated family physicians in the United States showed that the respondents were most prepared in the clinical aspects of patient care.^24^ It would appear that the Medical Expert objectives were ranked highly because of the clinical autonomy implied in the objective statements.

“Independently dealing with managerial and administrative aspects of a clinical practice” was ranked highly and similarly many qualitative suggestions revolved around managerial tasks. Literature shows that many new attending physicians feel more comfortable with their clinical skills in comparison with their non-clinical skills.^2,3,25,26^ A survey of 60 newly appointed emergency medicine physicians found that 63% of respondents felt the least confident in managerial issues.^4^ Almost every respondent in a survey of recently appointed pediatricians felt ill-prepared to cope with the non-clinical aspects of their practice.^27^ Seventy-five percent of newly appointed geriatricians advocated for more managerial training in residency.^28^ While our quantitative data did not rank non-clinical skills as the most important objective, our qualitative data suggests that respondents feel managerial and other non-clinical skills are very important to develop prior to beginning independent practice. This discrepancy may exist because, although it is felt to be very important that graduating residents master their clinical skills, many graduating residents may have achieved competency in these clinical areas and may actually need more training in the non-clinical skills which they have not yet mastered.

The importance of professional development is stressed in both the quantitative and qualitative data and implies a need to customize the TTP rotation for each resident’s professional goals. Residents will be encouraged to develop a personal learning plan to foster their own professional development with the help of faculty mentors and medical education literature on skill acquisition.^29^ Work-life balance and time management skills were suggested as potential TTP objectives that were not included in the quantitative survey.

Although “Completing a research, administrative, educational, or clinical project” received a low rank, there were many suggestions of project topics to be completed during the rotation. Many of the suggested projects involved protocol development, which represents an area of staff practice that is rarely emphasized in residency. In addition, there was a focus on projects involving medical education which was not highlighted as an important component of the TTP rotation. We feel that resident training does not prepare residents well for administrative tasks such as program and protocol development, and we intend to encourage completion of a project emphasizing these as part of the TTP rotation.

Strengths of this study include a high response rate and qualitative data collection that allowed for more specific suggestions to be captured. Weaknesses include a small sample size and relatively high-level objective wording which may have been hard to interpret for the respondents. In addition, our study population was multidisciplinary, which may have skewed the results due to some respondents not being as familiar with the needs of a newly appointed staff Radiation Oncologist, the current structure of residency training, and the upcoming changes associated with CBD implementation. However, we feel that the use of a multidisciplinary group was a strength as it provided a diverse interpretation of the learning objectives. This survey was intended to provide practical feedback in developing a TTP rotation in our department specifically, and may not be directly applicable to other departments with differing models of clinical care delivery and residency program administration.

A trial TTP structure at Dalhousie University,implemented in the spring of 2018, allowed each resident to contribute substantially to their own proposed TTP rotation schedule. The results from this survey guided the structure of the program which included activities such as teaching junior residents, developing a personal learning plan, performing administrative tasks and more independent day-time call shifts and/or clinical work, communication skills training, resiliency training and independent projects (such as policy development, protocol review or educational material creation). A self-evaluation portfolio was created to encourage reflection and identification of learning needs prior to TTP schedule proposition. Future research could focus on the impact of implemented TTP programs, both locally and across Canada as CBD programs as initiated nationally.

### Conclusion

The quantitative and qualitative data from this multidisciplinary survey based on CanMEDS roles guided the implementation of a TTP rotation for PGY-5 Radiation Oncology residents at a tertiary care centre in Atlantic Canada. Our study suggested that focus during this rotation should be placed on clinical skills such as communication and independent medical expert skills as well as non-clinical skills such as managerial tasks. These results may be relevant to other training programs developing TTP experiences.
